# Impact of body-oriented therapy on executive abilities in children with computer game addiction

**DOI:** 10.1192/j.eurpsy.2021.1515

**Published:** 2021-08-13

**Authors:** N. Kiseleva, S. Kiselev

**Affiliations:** 1 Laboratory For Brain And Neurocognitive Development, Ural Federal University, Ekaterinburg, Russian Federation; 2 Clinical Psychology, Ural Federal University, Ekaterinburg, Russian Federation

**Keywords:** body-oriented therapy, computer game addiction

## Abstract

**Introduction:**

It is known that children with computer game addiction have a risk for development of deficit in executive abilities. It is important to develop effective approaches for helping children with this addiction.

**Objectives:**

The goal of this study was to reveal effect of body-oriented therapy on executive abilities in children with computer game addiction. Particularly we compared the efficacy of two methods of treatment (body-oriented therapy for children vs. conventional motor exercises) in a randomized controlled pilot study.

**Methods:**

16 7-year-old children with computer game addiction were included and randomly assigned to treatment conditions according to a 2×2 cross-over design. The body-oriented therapy included the exercises from yoga and breathing techniques. To assess the executive functions and attention in children we used 5 subtests from NEPSY (Tower, Auditory Attention and Response Set, Visual Attention, Statue, Design Fluency). Effects of treatment were analyzed by means of an ANOVA for repeated measurements.

**Results:**

The ANOVA has revealed (p<.05) that for all 5 subtests on executive functions and attention the body-oriented therapy was superior to the conventional motor training, with effect sizes in the medium-to-high range (0.42-0.80).

**Conclusions:**

The findings from this pilot study suggest that body-oriented therapy can effectively influence the executive abilities in children with computer game addiction. However, it is necessary to do further research into the impact of body-oriented therapies on children with this addiction.

